# Information behaviours of people with type 2 diabetes in Kuwait: a grounded theory study

**DOI:** 10.1186/s12875-024-02577-0

**Published:** 2024-09-04

**Authors:** Zainab Meer, Ebaa Al-Ozairi, Genevie Fernandes, Sruthi Ranganathan, Jay Patel

**Affiliations:** 1 Leeds Beckett University, Leeds, UK; 2 Al Dabbus Cardiac Centre Al Adan, Adan Hospital, Hadiya, Kuwait; 3 Clinical Research Unit, Dasman Diabetes Institute, Dasman, Kuwait; 4 Centre for Population Health Sciences, Usher Institute, University of Edinburgh, Edinburgh, UK; 5 School of Clinical Medicine, University of Cambridge, Cambridge, UK; 6 Faculty of Medicine and Health, University of Leeds, Leeds, UK

**Keywords:** Information behaviour, Information needs, Grounded theory, Diabetes, Kuwait

## Abstract

**Background:**

Relative to country-specific epidemiological trends, Kuwait experiences a far greater burden of type 2 diabetes among its population. Information behaviours form a significant component of self-care management for patients diagnosed with type 2 diabetes, however this remains an understudied aspect of disease management. This study aims to investigate the information behaviours of patients with type 2 diabetes in Kuwait, and characterise the methods employed to manage their disease.

**Methods:**

This qualitative study employed a grounded theory method. Semi-structured interviews were conducted with twenty-seven participants over three phases of data collection in primary, secondary and tertiary healthcare settings across Kuwait. These were complemented by in-depth interviews to detail the information behaviours of these participants. The interviews were translated where appropriate, transcripts, and analysed through qualitative coding to synthesise the information behaviour patterns.

**Results:**

The findings demonstrated that living with type 2 diabetes involved a range of developmental and transformative stages, including changes to the patients’ emotional state, reconstruction of their lifestyle and identity, and changes in the ways they find and use information. Living with the chronic condition was viewed as a dynamic and transitional process, where patients’ information behaviours continually changed throughout the process across various identifiable stages. This dynamic pattern was reflected most prominently across the participants’ behavioural needs, sources and information-seeking patterns.

**Conclusion:**

Patients with type 2 diabetes continuously adapted their information behaviours to optimise the self-management of their condition across a relatively predictable pattern. Greater understanding of these behaviours across a wider population would improve the provision of clinical care for patients with diabetes.

**Supplementary Information:**

The online version contains supplementary material available at 10.1186/s12875-024-02577-0.

## Background

Type 2 diabetes is a major global public health issue, imposing a substantial impact on human health, and socioeconomic development. The Global Burden of Disease Study 2021 estimated that 529 million (95% uncertainty interval 500–564 million) lived with diabetes in 2021, with the highest age-standardised prevalence rates in the Middle East and North Africa (MENA) region (9.3% [8.7–9.9%]) [1]. Due to population growth and ageing, the disability adjusted life-years (DALYs) from diabetes have risen by 682.3% (600.7–758.0%) in Kuwait from 1990 to 2021, or 60.8% (44.7 to 77.9%), when considering the age-standardised DALY rates over the same period [1].

Given that this chronic condition is not curable, and management typically aims to elicit optimal glycemic control, the ability for patients to self-care is an essential component of diabetes care [2]. Self-management may include periodic monitoring of blood glucose, insulin use, regular exercise, following a personalised diet plan, and other medicines use. Several studies show the importance of self-care in the effectiveness of diabetes control [3–6], where higher levels of patient adherence to self-management activities is associated with improved glycemic control, positive psychosocial outcomes, positive lifestyle influences, and the prevention of diabetic complications [7–9]. Few papers contextualise the self-care of this chronic condition across nations in the Middle East, where the burden of disease is most pronounced.

Information behaviour concerns individuals’ information needs, information searching behaviours and subsequent use of information [10]. Therefore, information behaviour in the context of health, concerns how individuals engage and interact with information and the reasons underpinning these patterns, and studies their processes for findings, processing and using information. This understanding represents a central component of self-management for chronic health conditions. The information behaviours of specific groups, in the context of an informational need can be summarised through visual models. As the diagnosis of diabetes typically involves a process of information acquisition to learn to manage and co-exist with the disease, applying information behaviour research would enable an enhanced comprehension of the mechanisms that patients use to select, seek, and use information [11]. Qualitative research studies applying grounded theory methodologies to people living with diabetes have focused on specific demographics, disease types and geographical locations. For example, a recent study exploring the experiences of Palestinian adolescents with type 1 diabetes showed that adolescents had maladaptive experiences, with fears and worries about the unexpectedness of their future life, which was not sufficiently addressed or supported by healthcare workers [12]. A Canadian study of adults with type 2 diabetes described a three-phased process of integration, where patients would process having diabetes, reach a turning point where the diagnosis can not be denied, and accept that diabetes is a part of their lives [13].

A qualitative research approach enables clinicians, healthcare leaders, and patients to better understand the information behaviours of people with type 2 diabetes. Grounded theory methodology is a highly useful method for systematically characterising complex thematic concepts, and interlinkages and summarising these into usable theoretical frameworks. This study aimed to investigate the information behaviours of patients with type 2 diabetes in Kuwait.

## Methods

We applied a qualitative, grounded theory method. This methodology enabled the research team to consider broad, exploratory questions that would develop a multifaceted framework. Given the anticipated intricacies of connecting multiple patient perceptions in a single theoretical model, grounded theory allowed the researchers to constructively account for the full range of ways that patients learn to manage and co-exist with their disease, rather than attempt to silo these into narrow domains. In addition, this method requires a systematic qualitative approach facilitating inductive and iterative data collection over the study period.

### Study settings and participants

Participants were recruited from four study sites in Kuwait representing three levels of patient care: two primary healthcare settings (primary care), one general hospital (secondary care), and one dedicated diabetic research centre (tertiary care). A purposive, convenience sampling technique was initially applied to select these settings, which represented a representative range of participants with different education and socioeconomic contexts. As participants referred the lead researcher (ZM) to other adult participants with type 2 diabetes, the method adapted into a snowball sampling technique.

Inclusion criteria were: people diagnosed with type 2 diabetes; adults aged 18 years and above; Arabic or English speaker; and able to consent to participle in the study. The study sample consisted of 22 adult patients and 5 healthcare providers recruited through purposive, convenience sampling from the study settings described. Participants were informed that they were free to decline participation with no implications for their ongoing care provision. All individuals who received information on the study agreed to participate, and were not financially compensated for their participation. Interviews were conducted online via Skype (Windows Desktop version 8.113.0.210), and face-to-face at the participants’ homes or healthcare settings. ZM gained consent to audio record the interviews, and made notes during the interview and on reviewing the recordings.

### Data collection

In-depth, semi-structured interviews with all participants were conducted by ZM. An interview guide and brief demographic questionnaire, guided by themes from a preliminary literature review, was developed by the interviewer in English and Arabic (supplementary file). The literature review highlighted three key areas: diabetes experience; diabetes self-care management; and information behaviour related to self-care management [14–20]. The interview guide was designed to encourage the participants to explain their experiences through a structured narrative whilst allowing the interviewer the freedom to follow relevant lines of enquiry [21–24]. The demographic questionnaire enabled a more detailed comprehension of the participants’ sociodemographic characteristics relevant for further analysis.

### Data analysis

All interviews were recorded, and later transcribed. Native language interviews were also translated to English, and independently verified by a bilingual researcher (ZM) to ensure accuracy in the translated transcripts. Accurate translation of interviews is a central challenge in grounded theory studies, as any modification to the tone or message risks compromise to the integrity of the interviews. Achieving a valid translation and maintaining conceptual equivalence of the interview was achieved by using a competent bilingual translator, with awareness of relevant medical terminology, as advised for qualitative studies [25]. The research team regularly met to discuss key themes emerging from the interviews, and to refine the coding framework. A preliminary concept map was developed to visually represent the inter-relationships between various concepts and categories.

**Fig. 1:**
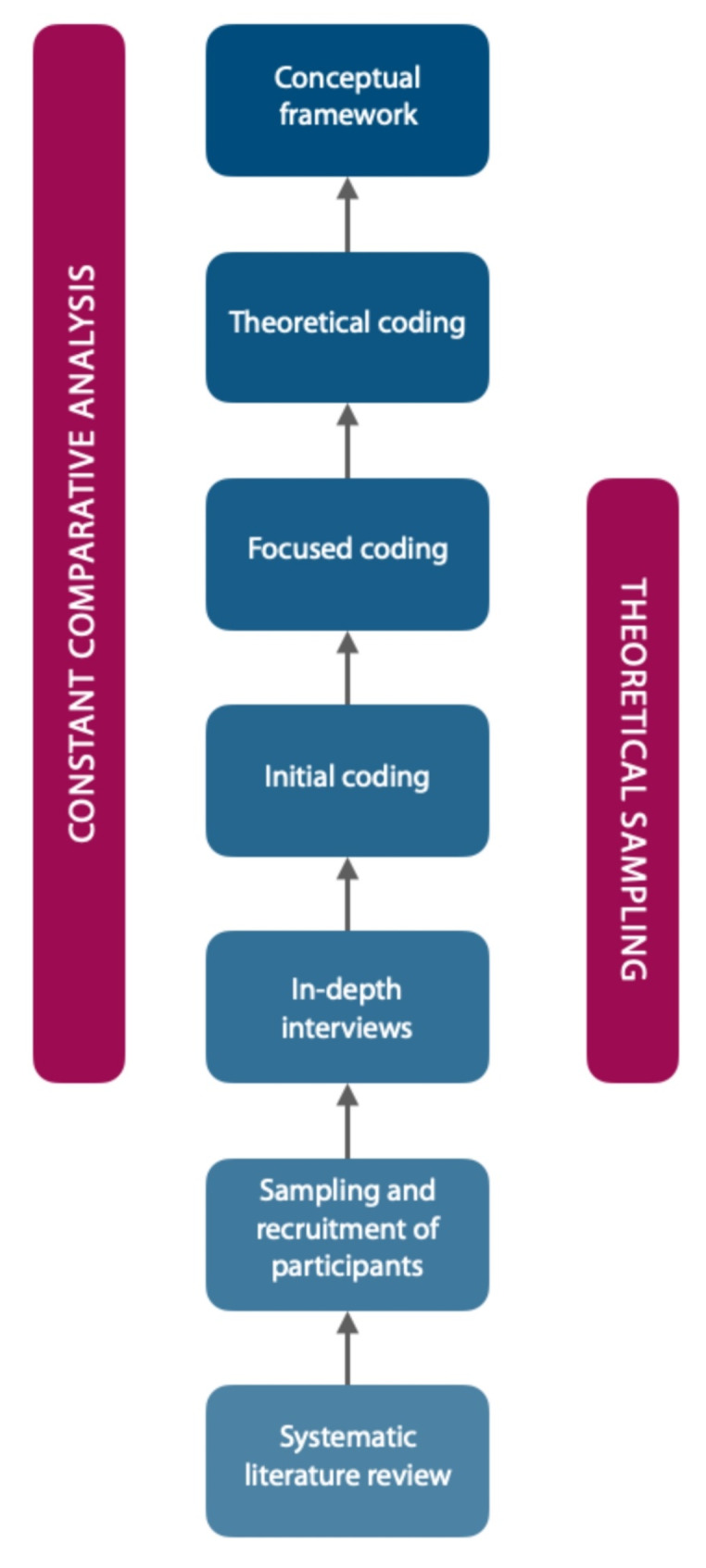
Schematic methodological overview of the grounded theory methodology used in this study. Adapted and modified from Charmaz, 2006

Coding was conducted across three phases: initial coding, focused coding and theoretical coding [22]. The coding strategies, memo writing, and constant comparative analysis assisted in the identification and construction of categories and concepts, and the development of the substantive theory, for representation as a schematic conceptual framework [21, 23]. The substantive theory was developed iteratively, beginning with initial codes generated in the initial stage, which were clustered into groups of focused codes. Focused codes were then grouped into a higher level of categories, enabling further theoretical sampling and analysis, until a core concept and substantive theory was produced. Further data analysis––through researcher (ZM) synthesising the qualitative insights to elicit key themes and linkages, comparative analysis and theoretical coding––was conducted to develop the conceptual framework. To execute the constant comparative analysis, findings were compared with previous interviews until no further data additions that contributed substantially to the key thematic concepts were ascertainable. Theoretical coding was applied to compare the findings with the literature, to identify new, or more appropriate thematic categories, or to identify linkages between existing themes. This also provided an insight into the current academic reasoning around diabetes information behaviours, and how these findings from Kuwait would improve the evidence base. Figure 1 summarises the methodological process used in this study.

## Results

This grounded theory study involved an analysis of information behaviours against a conceptual framework devised to represent the transitional phases navigated by patients with type 2 diabetes. The study identified temporal factors associated with information behaviour. Namely, experiencing unusual symptoms; acceptance of the diabetes diagnosis; adopting management strategies; adhering or relapsing; and adapting. Many participants expressed an educational approach that consisted of learning how to co-exist peacefully with the disease, in addition to adhering to the biomedical disease management.

### Phase 1: experiencing unusual symptoms

A phase of experiencing atypical symptoms was commonly cited as the first relevant stage in the information behaviour of patients with type 2 diabetes. Many participants noted differences in their physiological state of health, but were often unaware, or uncertain of the relevance of the symptoms to the disease.

For example, one participant unknowing of the symptoms of diabetes prior to their diagnosis assumed an alternative aetiology:

“I did not feel comfortable, I felt there was something wrong, but I was not sure if it was a sign of diabetes […] I was going to the toilet and urinating a lot. I thought this symptom was normal as I am growing up and becoming old”. (C: A7)

Even those with a family history of diabetes, and access to information about the condition were not confident in attributing a relationship between the symptoms and the disease. Some expressed their desire for further information on the clinical features of the disease, and their complications, to identify when to seek medical advice, and for better preparedness for the diagnosis, illustrated below:

“If I have had information about the symptoms, I would have been monitoring myself earlier and would have been ready.” (C: B13).

To better comprehend the symptoms, those concerned often sought opinions from family, friends and through internet searches. The interviews also elicited that many participants did not choose to seek information immediately after symptom onset, and waited to assess whether these were self-limiting, and attributable to a short period of feeling unwell.

“When I first experienced the symptoms, I did not search about it right away but then I tried to search some information from the internet though I was not sure, so I decided to seek medical consultation.” (C: B2).

### Phase 2: accepting the diagnosis

Following a professional diagnosis of type 2 diabetes, participants identified a desire for information, but some were unsure of what questions to ask, some required information to be able to co-exist with the condition, and some required information to enable a reconstruction of their self-identity and assist with life choices. The diagnosis led many to conflicting emotions, rendering some patients without the psychologically readiness to learn about the condition, and others confused about what information they required at this stage.

“In the beginning there was a lot to learn, but you will still be not ready to know and don’t know what to look for and what to ask.” (C: A4).

Gradually, participants accepted the role of diabetes in their daily lives, and were better able to define their information needs. Information that helped the patients cope with the disease, to alleviate fears from gaps in their knowledge, and for a better sense of management strategies prevailed as the most important needs. The reconstruction of self-identity involved the expression of difference, and in cases, abnormality from society, hence information seeking also provided consolation re-adjust the identity.

“Being diagnosed with diabetes made me think that I am different person now and I need to learn more of what this new person needs from now on to manage and co-exist with this disease.” (C: B8).

Health care professionals, family, friends and relatives were the most common points of contact for information after diagnosis. While healthcare practitioners were the first choice of contact, many participants were not able to address all their queries within allocated consultations, hence regular social contacts were frequently consulted. Generally, information was gradually sought following the diagnosis actively, and passively. The phase of accepting the diagnosis often elicited emotional stress, with some patients in denial, preventing their ability to muster the focus to locate and use information relevant to the condition. One participant vividly expressed the emotional impact of receiving their diagnosis on their information behaviours:

“Since I was diagnosed with diabetes, I did not take any medicine straightaway. I did not believe that I was diabetic! At that time, I couldn’t listen to the doctor’s instructions or look for any information.” (C: B8).

The account of other participants implied a momentary denial or disbelief, and.

“I stayed for a while before accepting the idea of being diabetic.” (C: A7).

Healthcare professionals, of varying types remained the primary point of contact for information, as highlighted in the accounts of three patients:

“After I accepted the reality of having diabetes, I started searching for information by asking my doctor, as I had some fears.” (C: B3).

“After being diagnosed with diabetes, I received some information from the nutrition specialist.” (C: B13).

“I used to receive most of information from my doctor in the clinic.” (C: B8).

### Phase 3: adopting management strategies

Common themes were developed within this phase, namely the need for simple, accessible information to understand practical aspects of diabetes self-care management and to help clarify uncertainties. As many participants had gained a baseline awareness of the disease through the prior phase, the information needs evolved to become more specific, shaped by their personal interests and needs, and aiding the acquisition of more tailored management strategies. Pharmacological management (drug types, benefits, dosage, usage, and side-effects), and the optimisation of lifestyle choices (diet and exercise) emerged as areas of high informational needs.

The sources of information for this phase expanding on those trusted for the diagnostic acceptance phase, included other patients with diabetes, television and social media, and religious leaders, in addition to healthcare professionals, family and friends. The expansion of information sources represented a more heterogeneous range of sources.

“I get most of the information I need from my doctor. Sometimes I ask my friends who are diabetic about what type of food they eat. I also get information from WhatsApp, especially about treatments and herbal recipes. Most of the time, I do not trust this information and I don’t follow them.” (C: B3).

Friends and faith groups were frequently sought to clarify contradictory aspects of self-management. Conflicting advice and differences in medical counselling from healthcare providers were not well received by participants, who preferred to have clearly-defined management approaches conveyed to them. Differences may have arisen, where more debatable aspects of diabetes care, lacking sufficient evidence to profess a single solution were discussed. This led to the concept of ‘doctor shopping’ being practised among some participants, who would discuss the varied advice with other diabetic patients, in the search for a healthcare professional who had a seemingly better ability to meet the patients’ information needs. The accounts of the three participants below, illustrate this.

“Sometimes I get confused about some of the contradicting information I have received from my doctors, so I only ask and seek this information from my friends.” (C: B2).

“One thing which annoys me is when doctors provide conflict information; I go to one doctor and he tells me that I have to take tablet for my diabetes, I go to another doctor and he tells me to take insulin injection. It took me a while to understand the best treatment option for my situation.” (C: B13).

“Every doctor will tell you something different on how to deal with diabetes. When I find it confusing, I ask my friends.” (C: B3).

Once the participants had accepted and processed their diagnosis, they began to seek information for self-care management strategies, including information seeking behaviours that were either active, passive or involved an avoidance approach. Information sought or received from other sources was largely applied in the context of optimal self-care management approaches, and methods to gain control of the disease. Two examples of active and passive information seeking behaviours are below:

“I started to read some leaflets given by the clinic and ask my doctor when I wanted to know anything […] I search online to get certain information I need. This helped me to learn about how to take care of myself and control my sugar levels.” (C: B3).

“I got messages through WhatsApp…I also received information about diabetes from family members and friends, particularly when I became confused with information provided by my doctor.” (C: A4).

Collaborative information seeking was a commonly observed pattern, where patients would seek the companionship of others to locate relevant information, or delegate the responsibility of retrieving information to close family members.

### Phase 4: adherence and relapse

Discussions with participants demonstrated that the diagnosis brought a sense of novelty to their lifestyle. As patients lived with the chronic disease over a prolonged period, they became increasingly disinterested with continued information seeking for their daily self-care management routine. The information behaviour patterns evolved to become largely characterised as information needs to minimise distress and to raise motivation. Self-care management was reported to become tedious, routine and boring, with several participants highlighting that they had become more care-free, returning to their previous, unhealthy habits and abandoned their diet and medication regimes. In this phase, only information behaviours with the capacity for modifying their self-care routine, minimising distress, and encouraging motivation were sought and used. For example.

“The matter has become a boring routine – even in the clinic; nobody tells you anything except repeating what they always tell you in each clinic visit and dispensing your monthly medicine. I need someone to give me any piece of information on how to release my stress or give me anything on how to change my tedious self-care routine.” (C: A4).

“I haven’t been ok in the last few months since I was uncommitted to the medication due to my life tensions and pressures […] I felt it become a tiring course of treatment. At that time, I didn’t want the usual instructions from my doctor, I just wanted her to tell me how to release my stress and give me some ways to go back to my self-care routine.” (C: B8).

Generally, participants showed two contrasting trajectories in this phase: continuing adherence to a diabetes self-care management programme; or entering a state of relapse, where a self-management strategy had collapsed, and glycaemic control was compromised. Where patients relapsed, information from family, friends and healthcare professionals were identified as the only sources of information with the potential capacity to re-align their disease control priorities. An experience of a medical doctor successfully realigning a relapsing patient was documented, highlighting the plasticity of relapse for these patients, and the capacity for behaviours remodelling.

“My doctor helped me to regulate my sugar levels after my case became worse by encouraging me to change my diet and lifestyle and giving me new ways of performing exercising.” (C: B3).

Seeking and receiving emotional and informational support had the capacity for positive change. Another participant had a similarly positive experience in this phase, abetted by a family member.

“My daughter also helped me to relieve my stress and pressures. She was always motivating me to face the disease. My daughter studies nutrition. She is also a good source of information to organise my meals. She also encourages me, and we go walking together.” (C: B8).

Frequently, the apparent disinterest in sustaining information behaviours emanated from a sense of having retrieved all relevant information pertaining to the condition, and experiencing no further reasons to find and use information in further optimising their diabetes care.

“After all these years, I already become expert (laughing). I don’t need to read or search for anything as I know everything.” (C: A7).

Others responded with a more defeatist attitude, suggesting that they felt primarily controlled by the disease, for instance:

“It is diabetes that controls me. I don’t search for information now as no matter what I do or when I try to search anything about it, nothing will work.” (C: A4).

### Phase 5: adaptation

In the final phase, described as an adaptation, participants described their information behaviour while they adapted to their illness and their self-care management. The adaptive phase highlighted two key thematic areas: (1) transitioning towards living and co-existing with the disease; and (2) balancing their needs.

After a protracted period living with diabetes, sufficient knowledge pertaining to self-care had been harnessed. Little further information for diabetes control was required.

“By this time, I believe that patients know everything and know how to treat themselves. They instinctively know what they need and how to deal with their problems. In the beginning I was curious to know how to manage the disease. I needed to know what type of food I should have and how to calculate carbs and calories. I wanted to know about the medicine and the way I should take it. But later on, my needs have changed. I felt I wanted to learn more about how to live and co-exist with my illness more easily, like knowing about how to relieve my stress with all the pressures I have without affecting my blood-sugar levels.” (C: B8).

The concept of balancing needs emerged, in the context of needing to resume daily actives, while sufficiently managing diabetes, but disallowing the opportunity for the disease to excessively dictate lifestyle decisions. One participant expressed this balanced as follows:

“It is very difficult to live as a normal person and having diabetes at the same time. You cannot eat or do things like other normal people. I wish if I can find a way to eat what I like without affecting my sugar level.” (C: A7).

In this final phase, the primary source(s) of information had changed from external sources (such as family, friends and healthcare professionals, as identified in previous stages), to learning from personal experience and trialling various strategies and reflecting on their successes. This transition characterised a sense of personal accomplishment in the information needs, and the self-promotion towards a sufficient expertise in self-management.

“After living with the disease for so many years, I try to know the appropriate diet for me by daily self-experience. For example, my doctor keeps telling me to stick to five meals a day. But my sugar level remains high. So, I started to organise the meals by sitting specific times that suits my daily lifestyle and what works for me. I passed from an experience onto another until eventually found a pattern to adjust my sugar level.” (C: B8).

“In time, I learned to know my body. Sometimes, when I shed tears, they become viscous and then I know that my blood sugar level is high. I, believe, that the patient usually knows when he or she gets sick, and he is the one who knows how to treat oneself. He or she, instinctively, know what he or she needs and deals with it.” (C: B3).

Information seeking behaviours largely shifted towards passive or avoidant strategies in response to new or unusual symptoms. Participants had also developed an ability to screen information whether retrieved passively or actively, and felt better able to evaluate their veracity. This was also the predominant reason for reducing information seeking, as the same participant demonstrated:

“After spending long time with the disease, I feel I know everything, and I am used to the things I need do to manage my disease. Everything is the same. There is nothing more you can learn about it.” (C: B3).

## Discussion

This qualitative, grounded theory study aimed to comprehensively understand the complexities of information behaviours in patients with type 2 diabetes in Kuwait. In particular, the study demonstrated how these behaviours shaped their self-care management. Analysis through the lens of information behaviour enabled a unique perspective on diabetes self-management. In the context of our analysis, information behaviour considered the participant’s actions (or inaction) when confronted with information needs. Information needs, and information seeking-behaviours were also considered and investigated. This analysis strongly supported the notion that the information behaviour of participants was not static but constantly changing throughout the transitional phases, as determined by their evolving information needs.

Several studies have explored the experience of patients with type 2 diabetes, particular with respect to how patients cope with their diagnosis and their reaction [26–31]. This study expands on the available evidence through delineating the journey from the first symptoms, through to the holistic adaptation necessary for optimal diabetes control. It uncovers and offers a unique insight into the stages and processes of patients’ information behaviour and how they learn to manage their disease. This insight is useful to health professionals who can benefit from understanding the lived experiences of patients and the critical times when support and information interventions can best be offered.

This study exemplified the significance of investigating the lived experience and information behaviours of patients with type 2 diabetes during a transitional period. Transitions, as a research area, has attracted increasingly multidisciplinary research interests [32–35]. However, information-based science remains frequently siloed from other research areas, such as the medical sciences [36]. This study addresses this gap by demonstrating the commonalities between two disciplines.

Dynamic changes in information behaviours are closely associated with changes in thoughts, feelings, actions, and situations [37]. During the phase of accepting the diabetes diagnosis, patients experienced various feelings which were dynamic, moving from denial to acceptance.

The study raised interesting insights into the role of denial. The study revealed, in line with existing evidence, that denial impacted patients’ adherence to their pharmacological regime and their commitment to self-care management [38–41]. However, the current study further revealed that denial can also be an obstacle to positive information-seeking behaviours. It appeared that patients who denied their disease psychologically were not ready to learn about it until the reality of having the disease was accepted. Hence, health care providers should emphasise the significance of accepting the chronic condition, particularly when designing and delivering management interventions in the early stages of diabetes and chronic disease diagnosis.

Furthermore, this study highlighted changes in information use across the five phases [11]. Notably, the concept of self-identity reconstruction had not been previously identified in the literature, and represents a novel dimension of information needs in patients with type 2 diabetes. There is a need to addresses this gap in the literature more comprehensively, and establish linkages between self-identity, acceptance and information behaviour. Healthcare professionals should support diabetic patients navigating often substantial changes in their self-identity, through providing the tools and skills to better integrate with a new or altered identity.

This research contributes to an enhanced understanding of the personal circumstances and experiences of patients living with type 2 diabetes. The granularity of detail through semi-structured interviews enables a useful basis to improve the quality of clinical care.

### Limitations

This study has some limitations. The mainstay of these limitations are inherent in the grounded theory methodology. Academic typically highlight the complexity and resource-intensive nature of a thorough coding process, and often the risk of oversimplifying complex situations. However, this is both an advantage and limitation, depending on the proposed utility of the results.

The limited sample size was chiefly due to challenges elicited by the COVID-19 pandemic. This was somewhat overcome by the notion that grounded theory studies typically benefit from the richness, depth, and originality of insights, rather than the volume of interviews [22]. A larger sample size may have confirmed findings, or may have contributed towards an expanded substantive theory, with novel thematic areas. The expansion of participants would have enriched and expanded the sociodemographic characteristics, allowing data to be sought from a broader cross-section of society, aiding the extrapolation of findings to other locations. Further studies are required using larger sample sizes and across different locations to further qualify the information behaviours of people living with type 2 diabetes.

## Conclusion

A variety of information behaviours were attributable throughout a transitional process of patients learning to live with and self-manage type 2 diabetes in Kuwait. Information behaviours were not static, but highly dynamic across a continuum of transitional changes across five key phases. This dynamic nature was reflected across the participants’ information behaviour, needs, sources and patterns. Healthcare professionals would benefit from considering the specify information behaviours of patients with type 2 diabetes to better support them throughout their journey of living with the disease.

## Electronic supplementary material

Below is the link to the electronic supplementary material.

Supplementary Material 1

Supplementary Material 2

Supplementary Material 3

## Data Availability

All data are available.
