# Supplementary figures and images for: Information behaviours of people with type 2 diabetes in Kuwait: a grounded theory study
Source: BMC Prim Care. 2024 Sep 4;25:326. doi: 10.1186/s12875-024-02577-0 (PMC11373242; doi:10.1186/s12875-024-02577-0)

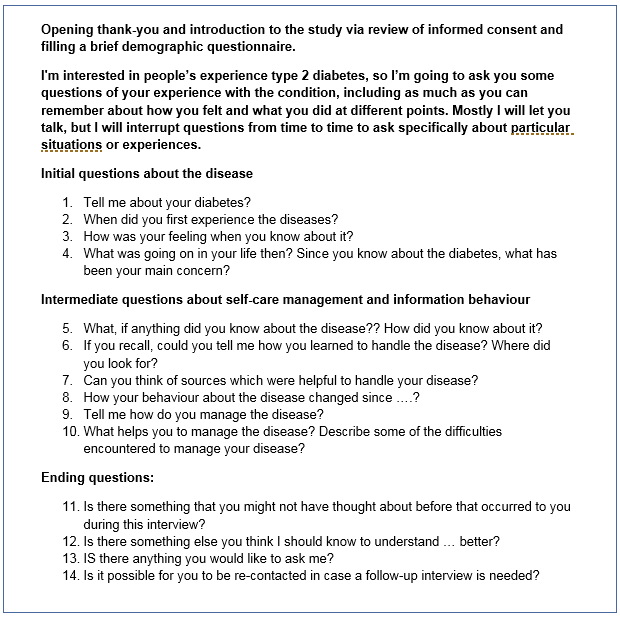
**Research Interview Guide**

**A Brief Demographic Questionnaire**
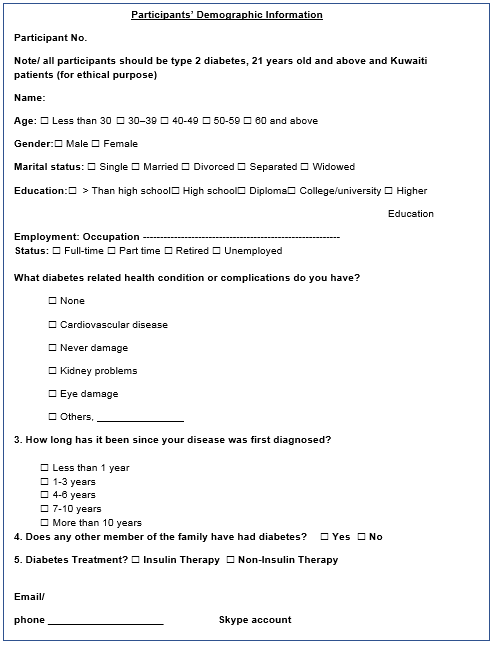

Supplement: Supplementary file 2 — Supplementary Material 2 [file 12875_2024_2577_MOESM2_ESM.docx]
