# Supplementary material for: Information behaviours of people with type 2 diabetes in Kuwait: a grounded theory study
Source: BMC Prim Care. 2024 Sep 4;25:326. doi: 10.1186/s12875-024-02577-0 (PMC11373242; doi:10.1186/s12875-024-02577-0)
Supplement: Supplementary file 1 — Supplementary Material 1 [file 12875_2024_2577_MOESM1_ESM.pdf]

9th December 2023

Dear Dr Goodridge,

I am delighted to submit a manuscript to *BMC Primary Care* as corresponding author, on behalf of my co-authors.

Manuscript: *Information behaviours of people with type 2 diabetes in Kuwait: a grounded theory study*

We believe the *BMC Primary Care* reflects the most appropriate outlet for the research team, reaching the receptive clinical, academic and industrial readership necessary to use the evidence to improve the lives of patients living with diabetes.

The paper provides a novel characterisation of the methods patients with type 2 diabetes use to manage their condition, exploring this robustly through a thematic analysis and presenting the findings using a conceptual framework. This framework subsequently forms the basis of a detailed grounded theory methodology, exploring these aspects in the context of information behaviours for patients living with type 2 diabetes in Kuwait. This paper mark a novel dimension of understanding for the management of type 2 diabetes in a country suffering a major health burden from the disease.

Our authorship represents an appropriate degree of diversity, with a mix of career stages, six academic and clinical institutions, gender representation, and the lead author from the study location (Kuwait).
All authors have approved the manuscript. We confirm that this work is original and has not been published elsewhere, nor is it currently under consideration for publication elsewhere. We have no competing interests to disclose. Please address all correspondence concerning this manuscript to patelj01@outlook.com.

Yours faithfully,

Jay Patel *on behalf of the authorship*

Dr Jay Patel
Centre for Population Health Sciences
University of Edinburgh
