# Supplementary material for: Information behaviours of people with type 2 diabetes in Kuwait: a grounded theory study
Source: BMC Prim Care. 2024 Sep 4;25:326. doi: 10.1186/s12875-024-02577-0 (PMC11373242; doi:10.1186/s12875-024-02577-0)
Supplement: Supplementary file 3 — Supplementary Material 3 [file 12875_2024_2577_MOESM3_ESM.pdf]

28 April 2024

Dr Goodridge
BMC Primary Care

Re: **Information behaviours of people with type 2 diabetes in Kuwait: a grounded theory study** [2bb5de0c-051c-4b52-9755-eb95e40cfebe]

Dear Dr Goodridge,

We thank you, and the reviewers for taking the time to consider our submission. We appreciate the constructive feedback and have addressed each of these below (in green) with corresponding alterations in the manuscript. We hope these modifications have improved the paper's suitability for publication, particularly ahead of the launch of the ***'Collection on Diabetes in Primary Care'***. Please do not hesitate to contact me, on behalf of authors, if any further clarifications are required.

Yours sincerely,

Dr Jay Patel
on behalf of the authorship

Reviewer Comments

Reviewer 1

GENERAL COMMENTS

- The topic is important and concerns a population of patients who are prevalent in the country. However, it is important to address in the introduction the importance of conducting this study, what already exist in the literature about this topic and what this study will add.

Thank you for taking the time to review the manuscript and for your constructive feedback. These points have been reviewed by the research team, and each comment has been addressed below, with corresponding amendments in the revised manuscript.

DETAILED COMMENTS:

ABSTRACT

- *Results section, page 2:* please elaborate to be more informative.

Thank you for your suggestion. We have expanded on this section. However, as this is the Abstract, we have retained brevity here.

- *Conclusion section: page 2, line 59*: "A wider populations" should be changed to 'a wider population"

Thank you for highlighting. This has now been altered.

INTRODUCTION

- It is important to address in the introduction the importance of conducting this study, what already there in the literature about this topic and what this study will add.

This is an inherent limitation of the literature, which does not contextualise research in the context of the Middle Eastern countries. We do appreciate that further context would be helpful for the reader. Hence, we expanded the introductory section to include further details around information behaviours in the context of health, and have included findings from other related studies.

- *Page 3, line 70*: Please remove "2" before the word "over".

Removed; thank you for highlighting.

- *Page 3, line 77*: Please remove the extra space after the first bracket of the ref: [7, 8, 9].

Amended, thank you.

- *Page 3, lines 78*-81: Please insert references from the relevant literature.

Amended, thank you.

- *Page 3, lines 85- 88*: Please insert references from the relevant literature.

Amended, thank you.

METHODS

o *In the methods section, please clarify the following points:*

1. I don't think that the snowballing technique is correct in describing the sampling strategy for this population in the current study. Healthcare settings in Kuwait with specialized diabetes clinics are known and distributed across the country based on governorates and health sectors. Therefore, you can base your sampling strategy by convenience or purposive.

Thank you for your feedback. We concur that this could be misleading, hence have expanded on this section to represent the evolution of sampling techniques applied. This begins with a purposive, convenience sampling method, which adapts into a snowballing technique.

2. Again, for participant sampling and selection, you need to be clear which sampling strategy was used; purposive versus convenience as both are different.

Thank you – this has been addressed above.

3. Why type 1 diabetes patients were excluded? Were there any exclusion/inclusion criteria for the healthcare providers?

The home-care management of type I diabetes is significantly different to that for type 2 diabetic patients. Therefore, this study focused only on type 2 diabetic patients, where lifestyle choices are a more prominent factor of their self-care. This aligns with the academic literature, where studies typically focus on one type of diabetes, rather than a conglomerate category.

- *Data collection section, page 4, line 114:* Please insert references from the preliminary literature review. The paragraph written in this section more describes the content of the interview guide not the data collection procedure. Please add a section in the manuscript "Study instrument" which should describe in detail the design and content of the study guide. In the data collection section, please give details describing the data collection procedure.

Thank you for your suggestion. We have now added the references from which findings were drawn from the literature review. We are happy to rephrase the heading as Study Instrument, but will do so on the basis of editorial discretion, as this represents a more stylistic alteration. Details pertaining to the interview guide have been expanded upon and the guide appears in the supplementary material. Data collection has also been expanded upon in the revised manuscript.

RESULTS

- There were no demographic data presented in the results section, regarding patient and healthcare provides.

Permission was not granted by the ethics committee in Kuwait to share demographic data for our patients due to differences in patient confidentiality protocols in Kuwait. These were respected.

- I would suggest having a diagram illustrating the conceptual framework that was generated from the data.

The conceptual framework appears in a separate manuscript by the research team. Generating the conceptual framework is not the core objective of this particularly study, hence to refine study aims, this has been omitted.

Figure 1: I would suggest deleting this figure and adding the PRR numbers to the tables next to each dimension.

Thank you for the suggestion. The authorship feel the figure is a useful representation of the methodological process. Hence, we will defer to editorial discretion on its inclusion or exclusion.

Reviewer 2

Thank you for the opportunity to review the manuscript titled "Information behaviours of people with type 2 diabetes in Kuwait: a grounded theory study" The manuscript is well-structured, however, there are a few areas that require further clarification and improvement.

Thank you for taking the time to review the manuscript and for your constructive feedback. These points have been reviewed by the research team, and each comment has been addressed below, with corresponding amendments in the revised manuscript.

Here are my comments:

Introduction:

It is not clear to me what information behaviours is. This statement "Information behaviour concerns individuals' information needs, information searching behaviours and subsequent use of information, therefore represents a central component of self-management for chronic health conditions." is very broad and so makes the aim very general. I am not sure what the authors are trying to investigate exactly. The background section could be further strengthened by including more recent studies on information behaviours and self-care management in diabetes, particularly from the Middle East region, to better contextualize the study.

Thank you for your comments. We appreciate that further context would be helpful for the reader. Hence, we expanded the introductory section to include further details around information behaviours in the context of health, and have included findings from other related studies. There are few studies pertaining to diabetes self-care in the Middle East, hence we have reverenced a paper from Palestine and Canada, as these were most relevant.

Methods: based on the general aim I am not sure if recruiting 5 healthcare providers would answer the study objective. If the aim was to reach saturation, this needs to be clearly stated. as I dont believe 5 healthcare providers would provide sufficient data to reach saturation. What was the inclusion criteria for the 5 healthcare providers?

We recognise that five healthcare providers is not the most ideal sample size. However, we were limited with resources, in terms of what was achievable in Kuwait. For these reasons, the five providers are of different care levels, and as representative of the Kuwaiti health system, as attainable. We have supplied further details on the purposive, convenience sampling methods.

In the data collection, you should cite the articles that were used for drafting the interview guide.

All relevant articles have now been cited in connection to the preparation of the interview guide.

For data analysis, explain further how the interviews were translated, who was involved? how was it validated?

We have expanded on this section to include a paragraph on translation and a recognition of its challenges in grounded theory studies.

The figure describes a starting point of systematic literature review, which is not fully described in text, more details are needed. I also suggest that the figure is flipped so the starting point is at the top or made from left to write.

We will follow editorial discretion on the arrangement of items in the figure. We chose an ascending view, since this is a hierarchical synthesis of evidence, beginning with the foundational systematic literature review, and culminating in the conceptual framework.

You can provide more details in the methods section:

Who was involved in conducting the interviews? you mention that some where conducted online, in patients home or in healthcare setting. Who selected the setting, who was involved in visiting the patients. how were the online interviews conducted. Was there anyone who was taking interview notes in addition to the interviewer? what is the experience of the researcher conducting the interviews? how did you ensure constency between arabic vs English interviews? was there a translation of the interview guide?

Thank you for these insights. We concur that these are methodological omissions and have addressed each point in the revised manuscript. We have significantly expanded on the section around translation.

The coding process needs to follow best practices related to this type of research. You indicate that codings were compared with literature, you should cite the literature you used.

All relevant articles have now been cited in connection to the coding strategy.

The results section is well-organized and presents the findings in a logical manner, following the five phases identified in the study: experiencing unusual symptoms, accepting the diagnosis, adopting management strategies, adherence and relapse, and adaptation. The authors have provided rich quotations from the participants to support their findings, which adds depth and credibility to the analysis.

Thank you for kind comments.

The authors have mentioned that the limited sample size was due to challenges posed by the COVID-19 pandemic. It would be helpful to discuss how this limitation might have impacted the study findings and the potential for further research with a larger sample size.

Thank you, this has now been added to the revised manuscript.

Minor comments:

Please ensure consistency in the use of the term "type 2 diabetes" throughout the manuscript.

Reviewed and amended throughout the manuscript for consistency.

Please review the reference list to ensure that all references are formatted consistently and contain complete information.

Reviewed and amended throughout the reference section for consistency.
